# Should trigeminal neuralgia be considered a clinically isolated syndrome?

**DOI:** 10.1177/13524585221149886

**Published:** 2023-01-26

**Authors:** Sarasa Tohyama, Jiwon Oh, Makenna Timm, Joshua C Cheng, Aisha Halawani, David J Mikulis, Andrew J Solomon, Mojgan Hodaie

**Affiliations:** Department of Physical Medicine and Rehabilitation, Spaulding Rehabilitation Hospital, Harvard Medical School, Boston, MA, USA/Division of Brain, Imaging and Behaviour—Systems Neuroscience, Krembil Brain Institute, Toronto Western Hospital, University Health Network, Toronto, ON, Canada/Institute of Medical Science, Faculty of Medicine, University of Toronto, Toronto, ON, Canada; Division of Neurology, Department of Medicine, St. Michael’s Hospital, Toronto, ON, Canada; Division of Brain, Imaging and Behaviour—Systems Neuroscience, Krembil Brain Institute, Toronto Western Hospital, University Health Network, Toronto, ON, Canada; Department of Neurology, Beth Israel Deaconess Medical Center, Harvard Medical School, Boston, MA, USA; Division of Brain, Imaging and Behaviour—Systems Neuroscience, Krembil Brain Institute, Toronto Western Hospital, University Health Network, Toronto, ON, Canada/Division of Neuroradiology, Joint Department of Medical Imaging, Toronto Western Hospital, University Health Network, Toronto, ON, Canada; Division of Brain, Imaging and Behaviour—Systems Neuroscience, Krembil Brain Institute, Toronto Western Hospital, University Health Network, Toronto, ON, Canada/Division of Neuroradiology, Joint Department of Medical Imaging, Toronto Western Hospital, University Health Network, Toronto, ON, Canada; Department of Neurological Sciences, Larner College of Medicine at the University of Vermont, Burlington, VT, USA; Division of Brain, Imaging and Behaviour—Systems Neuroscience, Krembil Brain Institute, Toronto Western Hospital, University Health Network, Toronto, ON, Canada/Institute of Medical Science, Faculty of Medicine, University of Toronto, Toronto, ON, Canada/Department of Surgery, Faculty of Medicine, University of Toronto, Toronto, ON, Canada/Division of Neurosurgery, Krembil Neuroscience Centre, Toronto Western Hospital, University Health Network, Toronto, ON, Canada

**Keywords:** Trigeminal neuralgia, multiple sclerosis, clinically isolated syndrome, diagnosis, diagnostic criteria, MRI, lesion

## Abstract

The association between trigeminal neuralgia (TN) and multiple sclerosis (MS) is well established. Many MS patients with TN have magnetic resonance imaging (MRI) evidence of a symptomatic demyelinating lesion. Although infratentorial presentations are included in the diagnostic criteria for MS, there remains confusion in clinical practice as to whether TN should be considered a clinically isolated syndrome for the application of McDonald criteria. In this case series, we discuss this diagnostic quandary in patients presenting with TN and additional MRI findings suggestive of MS and highlight the unmet need for data in such patients to optimally guide their care.

## Introduction

The association between trigeminal neuralgia (TN) and multiple sclerosis (MS) is well established.^1^ TN has been reported in 2%–10% of patients with MS^2–4^ and with an incidence 15-fold higher than in a general outpatient population.^4^ A pontine lesion proximal to the trigeminal ganglia is often, but not always, observed on magnetic resonance imaging (MRI) in patients with MS and TN.^4^ There remains disagreement in clinical practice regarding whether the presentation of a symptomatic central nervous system (CNS) lesion in the form of TN, accompanied by fulfillment of additional elements of the McDonald criteria (MC), is sufficient for the diagnosis of MS or whether such patients should instead be diagnosed with radiologically isolated syndrome (RIS). We present three patients with TN and demyelinating lesions suggestive of MS that highlight this diagnostic quandary and the unmet need for data to improve decision-making in such patients.

## Methods

Of the 481 patients who underwent neurosurgical treatment for TN at the Toronto Western Hospital in Canada between June 2004 and May 2018, we retrospectively identified three patients with a diagnosis of TN according to the International Classification of Headache Disorders-3,^5^ who also presented with MRI lesions highly suggestive of MS. All patients received longitudinal clinical and 3T MRI assessment approximately every 6 months or as needed as part of our routine follow-up care. An MS subspecialist neurologist (J.O.) reviewed all clinical records and MR images for evidence of events suspicious for demyelination and for 2017 MC^6^ fulfillment. The study was approved by the University Health Network Research Ethics Board.

## Cases

Demographic and clinical characteristics are summarized in Table 1.

**Table 1.** Demographic and clinical characteristics of three patients with trigeminal neuralgia and additional MRI lesions concerning for MS.

Case	Sex	Age at TN onset	TN duration (years)	Pain distribution	Clinical follow-up (months)	Imaging follow-up (months)	MR lesion presumed responsible for TN	MRI DIS	MRI DIT	CSF evaluation
1	F	36	13	Left V2/3	124	64	Yes	Yes (PV, I)	No	No
2	F	46	22	Left V2/3	87	90	Yes	Yes (PV, I)	No	No
3	F	44	25	Left V1/2/3	168	112	Yes	Yes (PV, I, JC, SC)	No	No

CSF: cerebrospinal fluid; DIS: dissemination in space; DIT: dissemination in time; F: females; I: infratentorial; JC: juxtacortical; MRI: magnetic resonance imaging; PV: periventricular; SC: spinal cord; TN: trigeminal neuralgia; V1: ophthalmic branch; V2: maxillary branch; V3: mandibular branch.

Clinical and MRI assessments were performed approximately every 6 months or as needed, as part of our routine follow-up care after neurosurgical treatment for TN.

### Case 1

**Figure 1.**
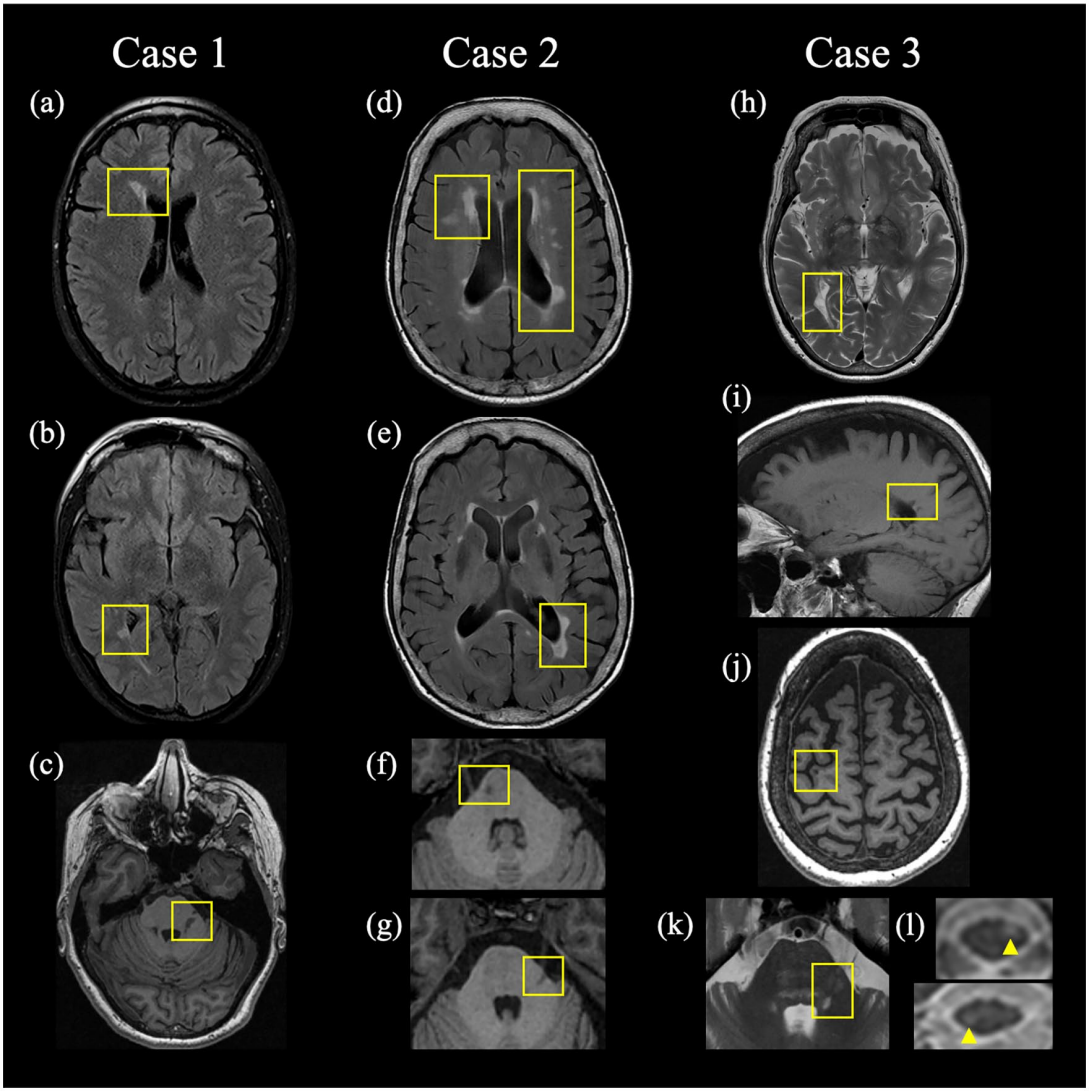
Magnetic resonance imaging sequences demonstrating demyelinating lesions characteristic of MS. Left column: axial T2-FLAIR MRI demonstrating periventricular lesions (a, b) and axial T1-weighted FSPGR MRI demonstrating a left-sided pontine lesion presumed responsible for TN (c) in Case 1. Middle column: axial T2-FLAIR MRI demonstrating periventricular lesions (d, e) and axial T1-weighted FSPGR MRI demonstrating infratentorial lesions (f, g), with the left-sided lesion (g) presumed responsible for TN in Case 2. Right column: axial T2-weighted and sagittal T1-FLAIR MRI demonstrating periventricular lesions (h, i), axial T1-weighted FSPGR MRI demonstrating a juxtacortical lesion (j), and axial T2-weighted MRI demonstrating the pontine lesion presumed responsible for TN (k) and spinal cord (l) lesions in Case 3. FLAIR: fluid-attenuated inversion recovery; FSPGR: fast spoiled gradient-echo.

A 49-year-old woman presented with a 13-year history of TN in the left V2/V3 distribution. She had no prior history of additional neurological symptoms. Neurological examination was normal. Brain MRI revealed a lesion within the dorsolateral left pons presumed responsible for TN, multiple T2 hyperintense supratentorial lesions concerning for MS (Figure 1(a)–(c)), and absent neurovascular contact with the trigeminal nerve. The patient was treated for TN and monitored clinically and radiologically and never received MS disease-modifying therapy (DMT). Ten years after presentation, neurological symptomatology remained restricted to TN and neurological examination remained unchanged. Brain MRI 5 years after presentation remained unchanged.

### Case 2

A 68-year-old woman presented with a 22-year history of TN in the left V2/V3 distribution. She had no prior history of additional neurological symptoms. Neurological examination was normal. Brain MRI revealed a left pontine lesion presumed responsible for TN, multiple T2 hyperintense lesions concerning for MS (Figure 1(d)–(g)), and absent neurovascular contact with the trigeminal nerve. The patient was treated for TN and monitored clinically and radiologically and never received DMT. Seven years after presentation, neurological symptomatology remained restricted to TN and both neurological examination and brain MRIs remained unchanged.

### Case 3

A 69-year-old woman presented with a 25-year history of left-sided TN in 2007 affecting V1–V3. She had no prior history of additional neurological symptoms. Neurological examination was normal. MRI revealed a pontine lesion presumed responsible for TN, multiple T2 hyperintense lesions concerning for MS including cervical and thoracic spinal cord lesions (Figure 1(h)–(l)), and absent neurovascular contact with the trigeminal nerve. The patient was treated for TN and was monitored clinically and radiologically and never received DMT. Fourteen years after presentation, neurological symptomatology had remained restricted to TN and neurological examination remained unchanged. Brain MRI remained unchanged 9 years after presentation.

## Discussion

We highlight the diagnostic challenge presented by TN in the setting of concurrent MRI evidence of CNS demyelination suggestive of MS. Diagnosis of relapsing-remitting MS^6^ can often be confirmed in patients presenting a clinically isolated syndrome (CIS) typical of MS—defined as an inflammatory event in the CNS developing acutely or subacutely with a duration of at least 24 hours.^6^ “Focal brainstem syndromes” are considered typical of MS by the 2017 MC, yet are not enumerated or described. Although paroxysmal symptoms are not mentioned in the 2017 MC, prior iterations have mentioned that such symptoms, if characteristic of MS, might be considered a relapse—but there are no further clarifying details. Should a paroxysmal syndrome, such as TN, be considered a CIS if there is objective evidence of a lesion?

While it is “typical” for MS patients to develop TN due to demyelinating lesions in the pons, such patients have not been included in many of the CIS cohorts^7,8^ that have informed MC. This differs from other brainstem syndromes such as internuclear ophthalmoplegia or cerebellar ataxia. Thus, while it may be reasonable to debate whether TN should be considered a CIS as defined by MC, there are likely insufficient data to reliably predict diagnostic accuracy for MS were MC applied in patients with TN—even if they also satisfied MC dissemination in time (DIT) and dissemination in space (DIS) and other diagnoses had been ruled out.

RIS is another diagnostic consideration for such patients. Yet, RIS typically describes the presence of MRI lesions highly suggestive of MS in patients with the absence of characteristic clinical signs and symptoms attributable to CNS demyelination—a characterization potentially inappropriate in patients with TN and corresponding infratentorial MRI-visible demyelinating lesions. RIS cohorts also typically have not included patients with TN. Consequently, it is not possible to apply longitudinal RIS data^9^ to assess risk of a future demyelinating attack or clinical progression in patients with TN and accompanying MRI lesions fulfilling DIS.

Novel data to justify the consideration of TN as CIS would influence treatment decisions and patient outcomes. In a recent study, TN preceded diagnosis of MS in 15% of patients.^3^ Similarly, in a prior large retrospective cohort, TN preceded MS in 14% of patients, with a range of 1–11 years before the next clinical symptom.^2^ In a different earlier cohort, TN was the first manifestation of MS in 3 of 22 (14%) patients.^10^ Data supporting application of MC in patients presenting with TN would therefore likely facilitate earlier diagnosis and treatment, which is associated with better clinical outcomes. Longitudinal data are needed—both the patients in this cohort and RIS studies^9^ demonstrate that not all patients go on to develop further demyelinating attacks or clinical disability associated with MS.

We note several limitations. The patients were seen at our neurosurgical center for refractory TN and not at symptom onset. Although careful history of prior neurological symptoms was obtained for each patient, including previous clinical history from external providers, our initial clinical and MRI assessment was on average 20 years from TN onset. Cerebrospinal fluid (CSF) evaluation was not performed. It is possible that patients did not recall mild symptoms that resolved, and the older age of presentation may have reduced the chances of radiological DIT in these patients.

Due to the dearth of adequate data in patients presenting with symptomatic infratentorial demyelinating lesions manifesting as TN and additional brain MRI lesions suggestive of MS, such patients presently defy diagnostic classification and present a diagnostic and prognostic challenge for clinicians attempting to navigate shared decision-making for treatment decisions. Given the close association between MS and TN, further studies involving cohorts of patients with TN and suspect MS are an important unmet need in the field.
